# Supplementary material for: Evaluating the potential of respiratory-sinus-arrhythmia biofeedback for reducing physiological stress in adolescents with autism: study protocol for a randomized controlled trial
Source: Trials. 2021 Oct 21;22:730. doi: 10.1186/s13063-021-05709-4 (PMC8530505; doi:10.1186/s13063-021-05709-4)
Supplement: Supplementary file 2 — Additional file 2. WHO items [file 13063_2021_5709_MOESM2_ESM.pdf]

| Data Category | Information |
|---|---|
| Protocol Acronym | RSA biofeedback in adolescents with autism |
| Primary registry and trial identifying number | ClinicalTrials.gov<br>Identifier: NCT04628715 |
| Date of registration in primary registry | November 13, 2020 |
| Source(s) of monetary or material support | Marguerite-Marie Delacroix Foundation |
| Primary sponsor | KU Leuven |
| Contact for public queries | Anoushka Thoen, PhD: anoushka.thoen@kuleuven.be, +3216372243, Herestraat 49 box 1510, 3000 Leuven |
| Contact for scientific queries | Prof. dr. Tine Van Damme: tine.vandamme@kuleuven.be, +3216326172, Herestraat 49 box 1510, 3000 Leuven<br><br>Department of Rehabilitation Sciences, Research Group for Adapted Physical Activity and Psychomotor Rehabilitation, KU Leuven, Leuven, Belgium and Department of Child Psychiatry, UPC KU Leuven, Leuven, Belgium |
| Public title | Evaluation of a biofeedback intervention to reduce stress in adolescents with autism spectrum disorder |
| Scientific title | Evaluating the potential of respiratory-sinus-arrhythmia biofeedback for reducing physiological stress in adolescents with autism: a randomized controlled study<br><br>Protocol Acronym: RSA biofeedback in adolescents with autism |
| Countries of recruitment | Belgium |
| Health condition(s) or problem(s) studied | Autism Spectrum Disorder |
| Intervention(s) | Supervised active intervention: RSA Biofeedback (1x/week, 5 weeks, 30 min/session and 20 min daily practice). The main goal during this intervention is to breath at resonance frequency. |
| | Supervised control intervention: sham RSA biofeedback (1x/week, 5 weeks, 30 min/session and 20 min daily practice). The participants will not practice at their resonance frequency, but at a normal breathing frequency during the sessions and during home practice. |
| | Non-supervised intervention: RSA biofeedback (1 guided session of 45 min, daily practice for 20 minutes, 5 weeks). The main goal during this intervention is to breath at resonance frequency. |
| Key inclusion and exclusion criteria | Ages eligible for study: 13-18 years;<br>Sexes eligible for study: both;<br>Recruitment of typically developing adolescents: yes, for cross-sectional phase |
| | Inclusion: Written informed consent/assent;<br>For adolescents with ASD only: Confirmed ASD-diagnosis (DSM-IV/5) |
| | Exclusion: Presence of intellectual disability (DSM-IV/5); Uncorrected hearing- or vision impairment; Presence of congenital heart diseases, diagnosed cardiovascular abnormalities or somatic diseases with a known impact on heart function; |

| | |
|---|---|
| | Pregnancy; Insufficient knowledge of Dutch language; Participation in other Clinical Trial(s);<br>For typically developing adolescents only: Presence of neurodevelopmental disorder or psychiatric disorder (DSM-IV/5); Autism Quotient score ≥32; Presence of a sibling with a neurodevelopmental disorder (DSM-IV/5) |
| Study type | Phase 1: Cross-sectional |
| | Phase 2: Interventional<br>- Stratified block randomization<br>- Parallel assignment during supervised intervention<br>- Participants are blinded for group allocation during supervised intervention<br>- No blinding procedures during non-supervised intervention<br>- Primary purpose: evaluation of efficacy and feasibility |
| Date of First Enrollment | November 26, 2020 |
| Target sample size | Adolescents with Autism Spectrum Disorder: 128 |
| | Typically developing adolescents: 38 |
| Recruitment status | Recruiting |
| Primary outcome(s) | Outcome name: Change in Root Mean Square of Successive Differences between normal heartbeats.<br>Method: time domain analysis measure of heart rate variability as measured by ECG.<br>Time points for typically developing adolescents: at baseline.<br>Time points for adolescents with autism spectrum disorder: at baseline; after 5 weeks supervised intervention; after 5 weeks non-supervised intervention. |
| | Outcome name: Logarithm of High-Frequency Heart Rate Variability (LnHF-HRV).<br>Method: frequency domain analysis measure of heart rate variability as measured by ECG.<br>Time points for typically developing adolescents: at baseline.<br>Time points for adolescents with autism spectrum disorder: at baseline; after 5 weeks supervised intervention; after 5 weeks non-supervised intervention. |
| Key secondary outcomes | Physiological assessment of heart rate, breathing frequency, skin conductance and fingertip temperature using a NeXus-10 MKII biofeedback device in combination with Biotrace+ Software. These measurements will be repeated at multiple assessment points (only for adolescents with autism spectrum disorder). In addition, change will be measured between the different tasks in both adolescent groups during the assessment itself.<br>Time points for typically developing adolescents: at baseline.<br>Time points for adolescents with autism spectrum disorder: at baseline; after 5 weeks supervised intervention; after 5 weeks non-supervised intervention. |
| | Cortisol level using three saliva samples during the assessment procedure for both adolescent groups.<br>Time points for typically developing adolescents: at baseline.<br>Time points for adolescents with autism spectrum disorder: at baseline; after 5 weeks supervised intervention; after 5 weeks non-supervised intervention. |
| | Behavioral data collected with<br>- Autism Spectrum Quotient - Adolescent Version at baseline assessment<br>- Social Responsiveness Scale - 2nd edition at baseline and after non-supervised intervention<br>- Repetitive Behavior Scale – Revised at baseline and after non-supervised intervention |

| | |
|---|---|
| | - Strengths and Difficulties Questionnaire (parent report) at baseline assessment and after the non-supervised intervention.<br>- Strengths and Difficulties Questionnaire (self-report) at baseline assessment and after the non-supervised intervention.<br>- Depression Anxiety and Stress Scale-21 items version at baseline, after 5 weeks supervised intervention; after 5 weeks non-supervised intervention.<br>- Perceived Stress Scale - Adolescent version at baseline, after 5 weeks supervised intervention; after 5 weeks non-supervised intervention.<br>- Visual Analogue Scale for stress perception during assessment at baseline, after 5 weeks supervised intervention; after 5 weeks non-supervised intervention.<br>- Visual Analogue Scale for Sensory hyper-responsivity at baseline, after 5 weeks supervised intervention; after 5 weeks non-supervised intervention.<br>- Physical Activity Vital Sign at baseline, after 5 weeks supervised intervention, after 5 weeks non-supervised intervention. |
| Ethics Review | Approved: 09/04/2021 (version 3.0)<br>Ethics Committee Research UZ/KU Leuven<br>Herestraat 49<br>B 3000 Leuven (Belgium)<br>Tel +32 16 34 86 00<br>ec@uzleuven.be<br><br>Approved: 20/10/2020 (version 2.0)<br>Ethics Committee Research UZ/KU Leuven<br>Herestraat 49<br>B 3000 Leuven (Belgium)<br>Tel +32 16 34 86 00<br>ec@uzleuven.be<br><br>Approved: 02/07/2020 (version 1.0)<br>Ethics Committee UPC KU Leuven<br>UPC Z.Org KU Leuven<br>Tel +32 27 58 05 10<br>ria.dhaeze@upckuleuven.be<br>Ethics Committee Research UZ/KU Leuven<br>Herestraat 49<br>B 3000 Leuven (Belgium)<br>Tel +32 16 34 86 00<br>ec@uzleuven.be |
| IPD sharing statement | No plan to share IPD |
| Protocol version | Issue date: 12/03/2021<br>Protocol version 3.0<br>Authors: AT, TVD, KA, JS<br><br>**Revision chronology:**<br>Protocol version 1.0, 17/06/2020 Original<br>Protocol version 2.0, 15/09/2020 Revision according to advice and recommendations of Ethics Committee Research UZ/KU Leuven<br>Protocol version 3.0, 12/03/2021 Amendment |
