# Supplementary material for: Evaluating the potential of respiratory-sinus-arrhythmia biofeedback for reducing physiological stress in adolescents with autism: study protocol for a randomized controlled trial
Source: Trials. 2021 Oct 21;22:730. doi: 10.1186/s13063-021-05709-4 (PMC8530505; doi:10.1186/s13063-021-05709-4)
Supplement: Supplementary file 3 — Additional file 3. Informed consent [file 13063_2021_5709_MOESM3_ESM.pdf]

INFORMATION AND CONSENT FORM

Title of the study: Evaluating the potential of respiratory-sinus-arrhythmia biofeedback for reducing physiological stress in adolescents with autism: study protocol for a randomized controlled trial

Work title part 1: Comparison study of stress parameters between adolescents with and without autism spectrum disorder

Work title part 2: The effectivity of a biofeedback intervention in adolescents with autism spectrum disorder

Name, address and contact information of the sponsor:
KU Leuven
Department Rehabilitation Sciences
Research group for Adapted Physical Activity and Psychomotor Rehabilitation
Herestraat 49 (O&N4, box 1510)
3000 Leuven (Belgium)

Name and contact information of the local investigator:

Prof. dr. Tine van Damme
Tine.vandamme@kuleuven.be

Prof. dr. Kaat Alaerts
Kaat.alaerts@kuleuven.be

dra. Anoushka Thoen
Anoushka.thoen@kuleuven.be

Prof. Dr. Jean Steyaert
Jean.steyaert@kuleuven.be

Central Ethical Committee:
Ethics Committee Research UZ/KU Leuven
ec@uzleuven.be

Information form

Dear parent(s)/representative(s),

We know that adolescents with autism often experience stress and previous studies have shown that the stress system in these adolescents works in a different way. Currently, a study at the KU Leuven has started that will further explore whether and how the stress system in adolescents with ASD (13-18 years) shows deviations compared to adolescents without ASD. The first part consists of a comparative study between adolescents with and without ASD and the second part contains an intervention study, about which you can find more information below.

For this study, we are still looking for adolescents with ASD. If you are interested in having your son/daughter participate in this study, we ask you to read the information below and to give your written consent since, in Belgium, parental consent is required for adolescents up to 18 years of age. This document consists of four parts: (1) essential information about the study; (2) the written consent for the parent/guardian for the young person; (3) information addressed to the young person; (4) the written consent for the young person

Background and aims of this study

Autism spectrum disorder (ASD) is a developmental disorder characterized by problems with social interaction, communication and the presence of certain stereotypical behaviors and interests. Many studies have been conducted to identify the causing factors of ASD and to better understand different behaviors of people with ASD. In recent years, several studies have been conducted focusing on the autonomic nervous system and its link to certain disorders such as ASD.

The autonomic nervous system is responsible for many processes in the body such as regulating heart rate, breathing, digestive processes and so on. These processes also include the regulation of the stress system. This system has two different tasks: on the one hand, it ensures that we take action when there is a certain threat, such as running away from a burning building. On the other hand, this stress system also ensures that we can achieve a state of rest afterwards. Some results of previous studies showed that there are differences in the functioning of the stress system between people with and without ASD and that these differences are also linked to certain symptoms and behaviors related to ASD. As a result, it was suggested by several researchers that these differences in the functioning of the stress system should be addressed by certain treatment methods. However, at this time, there is a need for more clarity regarding the differences in the functioning of the stress system of people with and without ASD before studies can be conducted to determine the effectiveness of certain treatment methods. Therefore, this study was divided into two parts.

In the **first part of this study**, we focus on investigating the functioning of the stress system of adolescents with and without ASD. For this purpose, adolescents between the ages of 13 and 18 will be examined using a stress test (60 minutes) that will take place on a campus of KU Leuven or at their school. Furthermore, some online questionnaires will be administered by the adolescents (30 minutes) and their parents (60 minutes).

The **second part of this study** will examine the effectiveness of a biofeedback intervention. This intervention allows physiological processes of which we are normally unaware, such as the heartbeat, to be represented visually on a screen. By applying certain breathing techniques, we try to influence the heart rate and in this way influence the stress system. This biofeedback intervention has already yielded positive results in several studies in other groups, such as a reduction in depressive complaints or a reduction in anxiety symptoms. A study has been conducted with children and adolescents with ASD, where positive results were also found.

The intervention that will be carried out in this study consists of two consecutive phases, each lasting 5 weeks. Thus, the total duration of the intervention is 10 weeks.

In the first phase, one 30-minute session, supervised by a researcher, will be offered weekly for 5 weeks at a campus of FaBeR (KU Leuven) or at the school of the adolescents. In addition, the adolescents will be asked to practice breathing at home for 20 minutes on the other days using an app on a smartphone. Instructions for this will be provided during the supervised weekly sessions. After each home training session, the adolescents will be asked via the app how the session went so that follow-up from a distance will be possible.

In the second phase, one session of 10 minutes will be supervised at a campus of FaBeR (KU Leuven) or at their school during which instructions will be given for the intervention that will be performed completely at home. For 5 weeks, daily sessions of 20 minutes will be proposed using an app on a smartphone and a wearable heart rate monitor (Polar). This will be worn around the chest during the sessions. After each daily session, the adolescent will be asked via the app how the session went so that some remote follow-up is assured.

If your son/daughter does not have a smartphone, the researcher will loan one. This will be handed back to the researcher at the end of the study.

After each phase, a test moment will be performed to verify the effectiveness of the previous phase. This test moment equates to the stress test from the first part of this study (the difference study) about which you can find more information below.

In order to examine the effectiveness of this intervention with sufficient reliability, a control intervention will also be conducted during the first phase. This is similar to the real intervention but will not contain the same effectiveness due to some modifications. The adolescents will be divided into these 2 intervention groups without knowing in which group they are. During the second phase, there will also be a control group but they will not receive an intervention.

Implications for the participant

Once you choose to have your son/daughter participate in this study, you and your son/daughter will be asked to complete some online questionnaires focusing on the presence of symptoms from the autism spectrum, certain behavioral characteristics, various aspects related to daily functioning, physical activity and the presence of stress. If severe psychiatric symptoms (major depression, psychosis, mania) and/or acute agitation are present, the participation of your son/daughter will be refused. If you would like more information on this subject, please feel free to contact the clinicians involved in this study (Prof. Dr. Jean Steyaert and Prof. dr. Tine Van Damme). If your son/daughter does qualify for the study, he/she will then be invited for the stress test, which takes one hour. This will take place outside school hours at a campus of FaBeR (KU Leuven) or at their school. During the stress test, two stress tasks will be performed and no invasive measurements will be used:

- 3 sensors will be applied around the fingers using Velcro and/or tape
- 1 stretchable band will be applied around the waist
- 3 self-adhesive sensors will be applied to the torso

In addition, three saliva samples will be collected throughout the test using a cotton swab on which the adolescent must chew for 1 minute. A summary of the duration and location of each part is shown in the table below.

Task	Duration	Location
Questionnaires for parent(s)/representative(s) about their child*	Max. 60 minutes	Home (online)
Questionnaires for the adolescent*	Max. 30 minutes	Home (online)
Stress test for the adolescent	Max. 60 minutes	Campus of FaBeR or at school
* These questionnaires must be completed and forwarded to the researchers before the stress test can be administered.		

The table below summarizes the different components of the second part of this study (the biofeedback intervention): its duration, the location of this intervention and the necessary materials. The stress test named below refers to the stress test from part 1 of this study, which was briefly explained above. The testing moment after phase 1 is combined with the supervised session of phase 2.

<table>
<tr><th>Part</th><th>Task</th><th>Duration</th><th>Location</th><th>Material needed</th></tr>
<tr><td rowspan="2">Phase 1:
5 weeks</td><td rowspan="2">Guided sessions with daily practice</td><td>Supervised sessions: 30 minutes</td><td>Campus of FaBeR or at school</td><td>Sensors as described during part 1 of this study</td></tr>
<tr><td>Daily practice: 4x5 minutes</td><td>Home</td><td>Smartphone and app</td></tr>
<tr><td rowspan="2">Testing after phase 1</td><td>Questionnaires for the adolescent*</td><td>Max. 20 minutes</td><td>Home (online)</td><td rowspan="2"></td></tr>
<tr><td>Stress test</td><td>Max. 60 minutes</td><td>Campus of FaBeR or at home</td></tr>
<tr><td>Phase 2: 5 weeks</td><td>Daily practice after 1 guided session</td><td>Supervised session: 10 minutes</td><td>Campus of FaBeR or at school</td><td>Smartphone, app and heart rate monitor</td></tr>
</table>

		Daily practice: 4x5 minutes	Home	
Testing after phase 2	Questionnaires for parent(s)/representative(s) about their child*	Max. 50 minutes	Home (online)	
	Questionnaires for the adolescent*	Max. 20 minutes	Home (online)	
	Stress test	Max. 60 minutes	Campus van FaBeR	
* These questionnaires must be completed and forwarded to the researchers before the stress test can be administered.				

<u>In case you would like to participate, you must know that:</u>

- Participation in this study is voluntary.
- You or your son/daughter have the right not to participate in this study.
- You or your son/daughter have the right to discontinue participation at any time. There is no need to provide a reason for doing so. The quality of your care will not be compromised as a result.
- The data that will be collected as part of this study will be treated as confidential. Your anonymity and the one from your son/daughter will be assured when the results are published. See below for more information.
- You and your son/daughter will be informed in time in case important new information is present that might affect your willingness to continue participating in this study.
- The investigator, the sponsor or the Ethics Committee UZ/KU Leuven can terminate your participation. This decision will be made for thorough reasons (e.g. safety of the participants). Permission from you, or your son/daughter as a participant, is not necessary for this.

<u>Benefits</u>

At the end of the study, your son/daughter can choose from some small reimbursements, such as two cinema tickets or a voucher worth 20 euros (bol.com), as a thank you for his/her participation in the study. If interested, you can also receive the general results of the study and the personal results of your son/daughter after completion of the study.

<u>Risks and inconveniences</u>

Any participation in a study involves a risk, as small as they can be. The sponsor is liable - even in the absence of fault - for the damage incurred by the participant or, in the event of his/her death, by his/her successors, and which is directly or indirectly related to his/her participation in the study. You or your son/daughter do not have to prove any fault in this respect. The sponsor has taken out insurance for this liability[1] (Amlin Insurance SE, policy number 299.053.700, Vanbreda Risk & Benefits NV, Plantin en Moretuslei 297, 2140 Antwerp). Except for the time investment, no discomfort is expected. However, should you experience any problems or discomfort, you may always contact the researchers of this study:

dra. Anoushka Thoen
anoushka.thoen@kuleuven.be

Prof. dr. Tine Van Damme
tine.vandamme@kuleuven.be

If you are confronted with psychological problems as a result of this study, you can always contact a clinician associated with this study. They will invite you for an interview.

Prof. dr. Tine Van Damme
tine.vandamme@kuleuven.be

Prof. Dr. Jean Steyaert
jean.steyaert@kuleuven.be

[1] These rights are defined by the European General Data Protection Regulation (AVG), by the Belgian Law on the Protection of Natural Persons with regard to the Processing of Personal Data and by the Law of August 22, 2002 on the Rights of the Patient.

Confidentiality of the data

All personal data collected for this study will be processed in an encrypted manner. This means that all participants will be assigned a unique number, which will be used to process the data. These numbers are not directly linked to phone numbers or email addresses. The contact details are only used for scheduling the stress tests, the supervised sessions of this study and any feedback on the results. You have the right to review these data and have corrections made if they are inaccurate .

A coding procedure is also used for the saliva samples similar to the one used for your medical data. The samples handed over to the sponsor are therefore only provided with an identification code in the context of this clinical study. The collected samples will be stored in and managed by UZ/KU Leuven Biobank. The manager of these samples (UZ/KU Leuven Biobank) commits to using these samples only in the context of this clinical study and to destroying them at the end of the stipulated storage period. The biological material is considered as a "donation" and you should be aware that in principle you will not receive any financial benefit (royalties) related to the development of new therapies resulting from the use of the biological material you donated that could have a commercial value. If you withdraw your consent to participate in the study, you can have your sample(s) destroyed or retrieved. To do so, contact the physician-investigator. Results obtained from your sample(s) before you withdrew your consent to participate remain the property of the sponsor.

All data collected for this study will be treated with utmost confidentiality according to the European General Data Protection Regulation (AVG/GDPR). In doing so, medical confidentiality, international guidelines (ICH-GCP) and Belgian legislation will be respected (including the legal requirements as stipulated in the EU Regulation 2016/679 (AVG) on the protection of privacy with regard to the processing of personal data and the Belgian Law of 22 August 2002 on patient rights). In addition, your personal data will be kept for 20 years within the research group and will be deleted afterwards. The collected saliva samples will only be kept within the research group during the study, afterwards they will be destroyed.

As the commissioner of this study, KU Leuven is the data controller of your personal data being processed in the context of this study. If you wish to discontinue your participation in the study, no further data will be collected. However, the data collected up to that point will be used for analysis. If you have any questions about how we use your data, you can always contact the principal investigator (Prof. dr. Tine van Damme) of this study.

If you have any questions about how we use your data or if you want to use your right to access, correct and, if necessary, stop further processing, you can always contact your investigator-doctor at the following contact address: jean.steyaert@kuleuven.be. If you have any further concerns or complaints, please contact the KU Leuven privacy team at privacy@kuleuven.be.

If you have any questions regarding your rights as a participant in the study, you can contact the Research Ethics Committee UZ/KU Leuven (ec@uzleuven.be; 016 34 86 00 (weekdays between 10 and 11 am).

Finally, you have the right to lodge a complaint about how your data is handled, with the Belgian supervisory authority responsible for enforcing data protection legislation:
Data Protection Authority (GBA)
Drukpersstraat 35,
1000 Brussels
+32 2 274 48 00
contact@apd-gba.be
https://www.gegevensbeschermingsautoriteit.be

Ethical committee

An independent ethics committee (Research Ethics Committee UZ/KU Leuven) approved this study. This study is performed according to the guidelines for good clinical practice (ICH/GCP) and according to the most recent version of the Declaration of Helsinki drawn up for the protection of people participating in clinical trials. Under no circumstances should you consider the approval by the UZ/KU Leuven Research Ethics Committee as an encouragement to participate in this study.

Contact

If you would like additional information and/or have questions regarding study participation, you can always contact dra. Anoushka Thoen or Prof. dr. Tine Van Damme by mail:

anoushka.thoen@kuleuven.be or tine.vandamme@kuleuven.be.

CONSENT FORM PARENTS / LEGAL REPRESENTATIVES

Title of the study: Evaluating the potential of respiratory-sinus-arrhythmia biofeedback for reducing physiological stress in adolescents with autism: study protocol for a randomized controlled trial

- □ As a parent/legal representative, I give permission for my son/daughter to participate in this study.
  - □ I have been in a position to read the information in this form and ask any additional information of the researchers. I have been free to choose whether or not to allow my son/daughter to participate in this study.
  - □ I have been informed that I am free to withdraw my participation at any time, without giving a reason.
- □ I, as a parent/legal representative, do NOT give permission for my son/daughter to participate in this study.

FIRST NAME SON/DAUGHTER ....

SURNAME SON/DAUGHTER ....

DATE OF BIRTH SON/DAUGHTER ....

Date, name and signature parent(s)/legal representative(s):

Upon completion of this study (please indicate):

- □ I would like to receive the overall results of the study.
- □ I would like to receive the personal scores on the questionnaires.
- □ I would like to receive the personal results of the stress tests.
- □ I would like to know in which group my son/daughter was placed.
- □ I would like to receive an overview of my son/daughter's progress throughout the study.

You may send this information to the following email address:

INFORMATION AND ASSENT FORM

Title of the study: Evaluating the potential of respiratory-sinus-arrhythmia biofeedback for reducing physiological stress in adolescents with autism: study protocol for a randomized controlled trial
Work title part 1: Comparison study of stress parameters between adolescents with and without autism spectrum disorder
Work title part 2: The effectivity of a biofeedback intervention in adolescents with autism spectrum disorder

Researchers:
Prof. dr. Tine Van Damme
dra. Anoushka Thoen
Prof. dr. Kaat Alaerts
Prof. Dr. Jean Steyaert

Information form

In recent years, researchers have been working a lot on finding out the cause of ASD (autism spectrum disorder) so they can develop new treatments that can help these people move forward. Some of this research is about how the stress system works. Everyone has such a system and normally it ensures, for example, that your heart beats faster when you are afraid but also that you can calm down as soon as you are no longer afraid. In people with ASD, researchers have found that the stress system of these people does not work quite the same as in people without ASD. However, in order to get more clarity on this, more studies would have to be executed. Therefore, we would like to ask you if you are interested in participating in this study that consists of 2 parts. In the first part, we want to examine whether there are differences in the stress system between adolescents with and without ASD. In the second part, we want to examine whether a certain treatment, which has already been used with other children and adults, works sufficiently for young people with ASD. During this treatment you will learn how to breathe in an adapted manner.

You are free to choose whether you want to participate in this research and you can always say if you no longer want to participate. If you decide to participate and your parents have also given their permission, you will be asked to carry out the following tasks:

For the first part of the study:

1) Completing questionnaires: both you and your parents will be sent some online questionnaires. You should try to complete these as completely as possible. For you, these questionnaires will take about half an hour. The questionnaires focus on the presence of symptoms from the autism spectrum, certain behavioral characteristics, various aspects related to daily functioning, physical activity and the presence of stress. If these show that you have severe psychiatric symptoms, you and your parents will be informed and your participation in the study will be refused. If you and your parents would like additional information, you can always contact the clinicians involved in this study (Prof. Dr. Jean Steyaert and Prof. dr. Tine Van Damme).

2) Carrying out a stress test: this will be carried out on a KU Leuven campus or at your school and will last approximately one hour. A researcher will explain the test to you gradually. During the test, the researcher will use some sensors (small measuring instruments):
   - 3 sensors will be attached with Velcro and/or tape around your fingers
   - 1 wide band will be applied around your waist
   - 3 self-adhesive sensors will be placed on your torso

In this way, the examiner can look at your heart rate and your breathing, among other things. Finally, the examiner will also ask you three times to chew on a cotton swab so that we can collect some of your saliva.

For the second part of the study:

1) Following a 5-week treatment: during this, you will have an supervised session together with a researcher once a week on a campus of KU Leuven or at your school that lasts 30 minutes. During this session, you will learn how to breathe in other ways. Next, the researcher will ask you to practice your breathing for 20 minutes every day. You can divide those 20 minutes into 4 times 5 minutes so that you can spread it throughout the day. For example, you can practice 2 times 5 minutes in the morning and 2 times 5 minutes in the evening. For this home exercise, you will be able to use an app that will support you in this.

2) Filling out questionnaires and performing a stress test, as described above in part 1 of this study. The questionnaires now take 20 minutes so they are a little shorter.

3) Following a treatment of 5 weeks: you will also practice with your breathing but the researcher will only go through 1 session with you, after that you will be asked to practice every day for 20 minutes with your breathing. You can divide these 20 minutes into 4 times 5 minutes so that you can spread them throughout the day. For this home exercise, you will be able to use an app that will support you. In addition, you will also use a heart rate monitor that you can wear as a band around your torso.

4) The completion of questionnaires by you and your parents and performing a stress test as described in step 2 above.

Benefits

If you wish to participate, you can choose from several small compensations, such as two cinema tickets or a voucher of 20 euros (bol.com), as a thank you for your participation. If interested, you and your parents can also receive the general results of the study and your personal results after completion of the study.

If you have any questions about this study, please discuss them with your parents. If necessary, you can also send an e-mail to one of the researchers:

dra. Anoushka Thoen
anoushka.thoen@kuleuven.be

Prof. dr. Tine Van Damme
tine.vandamme@kuleuven.be

ASSENT FORM

Title of the study: Evaluating the potential of respiratory-sinus-arrhythmia biofeedback for reducing physiological stress in adolescents with autism: study protocol for a randomized controlled trial

- □ I have been allowed to choose whether or not to participate in this study.
    - □ I want to participate in this study.
    - □ I have been given the opportunity to read this letter and to ask questions when something was not clear.
    - □ It is clear to me that I may stop at any time and that I do not have to tell anyone why I no longer want to participate.
- □ I do NOT want to participate in this study.

FIRST NAME ....

SURNAME ....

DATE OF BIRTH ....

Date, name and signature:

Upon completion of this study (please indicate):

- □ I would like to receive the overall results of the study.
- □ I would like to receive the personal scores on the questionnaires.
- □ I would like to receive the personal results of the stress tests.
- □ I would like to find out which group I was assigned to.
- □ I would like to receive an overview of my progress during the study.

You may send this information to the following e-mail address:

_ _
