# Supplementary material for: Evaluating the potential of respiratory-sinus-arrhythmia biofeedback for reducing physiological stress in adolescents with autism: study protocol for a randomized controlled trial
Source: Trials. 2021 Oct 21;22:730. doi: 10.1186/s13063-021-05709-4 (PMC8530505; doi:10.1186/s13063-021-05709-4)
Supplement: Supplementary file 4 — Additional file 4. Informed consent [file 13063_2021_5709_MOESM4_ESM.pdf]

INFORMATION AND CONSENT FORM

Title of the study: Evaluating the potential of respiratory-sinus-arrhythmia biofeedback for reducing physiological stress in adolescents with autism: study protocol for a randomized controlled trial

Work title: Comparison study of stress parameters between adolescents with and without autism spectrum disorder

Name, address and contact information of the sponsor:
KU Leuven
Department Rehabilitation Sciences
Research group for Adapted Physical Activity and Psychomotor Rehabilitation
Herestraat 49 (O&N4, box 1510)
3000 Leuven (Belgium)

Name and contact information of the local investigator:

Prof. dr. Tine van Damme
Tine.vandamme@kuleuven.be

Prof. dr. Kaat Alaerts
Kaat.alaerts@kuleuven.be

dra. Anoushka Thoen
Anoushka.thoen@kuleuven.be

Prof. Dr. Jean Steyaert
Jean.steyaert@kuleuven.be

Central Ethical Committee:
Ethics Committee Research UZ/KU Leuven
ec@uzleuven.be

Information form

Dear parent(s)/representative(s),

We know that adolescents with autism often experience stress, and previous studies have shown that the stress system works differently in these adolescents. Currently, a study is ongoing at KU Leuven which investigates if and how the stress system in adolescents (13-18 years old) with an autism spectrum disorder (ASD) shows deviations compared to adolescents without ASD. Because we are also looking for adolescents without ASD for this study, some schools, including yours, were contacted who are willing to further disseminate the data from this study.

Since parental consent must be obtained in Belgium for adolescents up to 18 years of age, we ask you to read the information below and to give your written consent if you are interested in having your son/daughter participate in this study. This document consists of four parts: (1) essential information about the study; (2) written consent for the young person's parent/guardian; (3) information addressed to the young person; (4) written consent for the young person.

Background and aims of this study.

An autism spectrum disorder (ASD) is a developmental disorder characterized by problems with social interaction, communication, and the presence of certain stereotypical behaviors and interests. Many studies have already been conducted to identify the causing factors of ASD and to better understand the behaviors exhibited by people with ASD. In recent years, several studies have also been conducted focusing on the autonomic nervous system and its link to certain disorders such as ASD.

The autonomic nervous system is responsible for many processes in the body such as regulating heart rate, breathing, digestive processes and so on. These processes also include the regulation of the stress system. This system has two different tasks: on the one hand, it ensures that we take action when there is a certain threat such as running away from a burning building and on the other hand this stress system also ensures that we can achieve a state of rest afterwards.

Some results of previous studies showed that there are differences in the functioning of the stress system between people with and without ASD and that these differences are also linked to certain symptoms and behaviors related to ASD. As a result, it has been suggested by several researchers that these differences in the functioning of the stress system should be addressed by certain treatment methods. However, currently there is a need for more clarity regarding the functioning of the stress system of people with and without ASD, before studies can be conducted to determine the effectiveness

of certain treatment methods. Therefore, this study focuses on adolescents with and without ASD to create more clarity on the different functioning of the stress system. For this purpose, adolescents between the ages of 13 and 18 will be examined using a stress test and some online questionnaires will be administered by both the adolescents and their parents.

Implications for the participant

Once you choose to have your son/daughter participate in this study, you and your son/daughter will be asked to complete some online questionnaires that focus on the presence of symptoms from the autism spectrum, certain behavioral characteristics, various aspects related to daily functioning, physical activity and the presence of stress. If these questionnaires reveal that symptoms from the autism spectrum are present in your son/daughter, you will be informed of this and your son/daughter's participation in the study will be refused. If you would like additional information, you can always contact the clinicians involved in this study (Prof. Dr. Jean Steyaert and Prof. dr. Tine Van Damme). If your son/daughter does qualify for the study, he/she will then be invited for the stress test, which takes one hour. This will be conducted at school or, if you wish, at a FaBeR campus at KU Leuven. During the stress test, 2 stress tasks will be performed and no invasive measurements will be used:

- 3 sensors will be applied around the fingers using Velcro and/or tape
- 1 stretchable band will be applied around the waist
- 3 self-adhesive sensors will be applied to the torso

In addition, three saliva samples will be collected throughout the test using a cotton swab that must be chewed for 1 minute. A summary of the duration and location of each part is shown in the table below.

Task	Duration	Location
Questionnaires for parent(s)/representative(s) about their son/daughter*	Max. 60 minutes	Home (online)
Questionnaires fort he adolescent*	Max. 30 minutes	Home (online)
Stress test for the adolescent	Max. 60 minutes	Campus of FaBeR or at school
* These questionnaires must be completed and forwarded to the researchers before the stress test can be administered.		

If you wish to participate in this study, you should know that:

- Participation in this study is voluntary.
- You or your son/daughter have the right not to participate in this study.
- You or your son/daughter have the right to discontinue participation at any time. There is no need to provide a reason for doing so.
- The data that will be collected as part of this study will be treated as confidential. Your anonymity and the one of your son/daughter will be assured when the results are published. See below for more information.
- You or your son/daughter will be notified in time in case important new information is present that might affect your willingness to continue participating in this study.
- The investigator, the sponsor or the Research Ethics Committee UZ/KU Leuven can stop your participation. This decision will be made for thorough reasons (e.g. safety of the participants). Permission from you, or your son/daughter as a participant, is not necessary for this.

Benefits

At the end of the study, your son/daughter can choose from a few small compensations, such as a cinema ticket or a 10-euro voucher (bol.com), as a thank you for his/her participation in the study. If interested, you can also receive the general results of the study and the personal results of your son/daughter after completion of the study.

Risks and inconveniences

Any participation in a study involves a risk, as small as they can be. The sponsor is liable - even in the absence of fault - for the damage incurred by the participant or, in the event of his/her death, by his/her successors, and which is directly or indirectly related to his/her participation in the study. You or your son/daughter do not have to prove any fault in this respect. The sponsor has taken out insurance for this liability[1] (Amlin Insurance SE, policy number 299.053.700, Vanbreda Risk & Benefits NV, Plantin en Moretuslei 297, 2140 Antwerp).

Except for the time investment, no discomfort is expected. However, should you experience any problems or discomfort; you may always contact the researchers of this study:

dra. Anoushka Thoen
anoushka.thoen@kuleuven.be

Prof. dr. Tine Van Damme
tine.vandamme@kuleuven.be

If you are faced with psychological problems as a result of this study, you can always contact a clinician associated with this study. They will then invite you for an interview.

Prof. dr. Tine Van Damme
tine.vandamme@kuleuven.be

Prof. Dr. Jean Steyaert
jean.steyaert@kuleuven.be

Confidentiality of the data

All personal data collected for this study will be processed in an encrypted manner. This means that all participants will be assigned a unique number, which will be used to process the data. These numbers are not directly linked to phone numbers or email addresses. The contact information will only be used for scheduling the stress test of this study and any feedback on the results. You have the right to review these data and have corrections made if they are inaccurate.

A coding procedure is also used for the saliva samples similar to the one used for your medical data. The samples transferred to the sponsor are therefore only provided with an identification code in the context of this clinical study. The collected samples will be stored and managed by UZ/KU Leuven Biobank. The manager of these samples (UZ/KU Leuven Biobank) commits to using these samples only in the context of this clinical study and to destroying them at the end of the stipulated storage period. The biological material is considered as a "donation" and you should be aware that in principle you will not receive any financial benefit (royalties) related to the development of new therapies resulting from the use of the biological material you donated that could have a commercial value. If you withdraw your consent to participate in the study, you can have your sample(s) destroyed or retrieved. To do so, contact the physician-investigator. Results obtained from your sample/s before you withdrew your consent to participate remain the property of the sponsor.

All data collected for this study will be treated with utmost confidentiality according to the European General Data Protection Regulation (AVG/GDPR). In doing so, medical confidentiality, international guidelines (ICH-GCP) and Belgian legislation will be respected (including the legal requirements as stipulated in the EU Regulation 2016/679 (AVG) on the protection of privacy with regard to the processing of personal data and the Belgian Law of 22 August 2002 on patient rights). In addition, your personal data will be kept for 20 years within the research group and will be deleted afterwards. The collected saliva samples will only be kept within the research group during the study, afterwards they will be destroyed.

As the commissioner of this study, KU Leuven is the data controller of your personal data processed in the context of this study. If you wish to discontinue your participation in the study, no further data will be collected. However, the data collected up to that point will be used for analysis. If you have any questions about how we use your data, you can always contact the principal investigator (Prof. dr. Tine van Damme) of this study.

If you have any questions about how we use your data or wish to use your right to access, correct and, if necessary, stop further processing, you can always contact your physician-investigator at the following contact address: jean.steyaert@kuleuven.be. If you have any further concerns or complaints, please contact the KU Leuven privacy team at privacy@kuleuven.be.

---

[1] These rights are defined by the European General Data Protection Regulation (AVG), by the Belgian Law on the Protection of Natural Persons with regard to the Processing of Personal Data and by the Law of August 22, 2002 on the Rights of the Patient.

If you have any questions regarding your rights as a participant in the study, you can contact the Research Ethics Committee UZ/KU Leuven (ec@uzleuven.be; 016 34 86 00 (weekdays between 10 and 11 am).

Finally, you have the right to lodge a complaint about how your data is handled, with the Belgian supervisory authority responsible for enforcing data protection legislation:
Data Protection Authority (GBA)
Drukpersstraat 35,
1000 Brussels
+32 2 274 48 00
contact@apd-gba.be
https://www.gegevensbeschermingsautoriteit.be

Ethical Committee

An independent ethics committee (Research Ethics Committee UZ/KU Leuven) approved this study. This study is conducted according to the guidelines for good clinical practice (ICH/GCP) and according to the most recent version of the Declaration of Helsinki drawn up for the protection of people participating in clinical trials. Under no circumstances should you consider the approval by the Ethics Committee Research UZ/KU Leuven as an encouragement to participate in this study.

Contact

If you would like additional information and/or have questions regarding study participation, you can always contact dra. Anoushka Thoen or Prof. dr. Tine Van Damme via e-mail:

anoushka.thoen@kuleuven.be or tine.vandamme@kuleuven.be.

CONSENT FORM PARENTS / LEGAL REPRESENTATIVES

Title of the study: Evaluating the potential of respiratory-sinus-arrhythmia biofeedback for reducing physiological stress in adolescents with autism: study protocol for a randomized controlled trial

□ As a parent/legal representative, I give permission for my son/daughter to participate in this study.

  □ I have been in a position to read the information in this form and ask any additional information of the researchers. I have been free to choose whether or not to allow my son/daughter to participate in this study.

  □ I have been informed that I am free to withdraw my participation at any time, without giving a reason.

□ I, as a parent/legal representative, do NOT give permission for my son/daughter to participate in this study.

FIRST NAME SON/DAUGHTER ....

SURNAME SON/DAUGHTER ....

DATE OF BIRTH SON/DAUGHTER ....

Date, name and signature parent(s)/legal representative(s):

Upon completion of this study (please indicate):

□ I would like to receive the overall results of the study.
□ I would like to receive the personal scores on the questionnaires.
□ I would like to receive the personal results of the stress tests.

You may send this information to the following email address:

INFORMATION AND ASSENT FORM

Title of the study: Evaluating the potential of respiratory-sinus-arrhythmia biofeedback for reducing physiological stress in adolescents with autism: study protocol for a randomized controlled trial

Work title: Comparison study of stress parameters between adolescents with and without autism spectrum disorder

Researchers:
Prof. dr. Tine Van Damme Prof. dr. Kaat Alaerts Prof. Dr. Jean Steyaert dra. Anoushka Thoen

Information form

Young people with autism spectrum disorder, also abbreviated as ASD, have difficulties communicating with other people and also exhibit other characteristics such as, for example, very limited interests in certain things. In recent years, researchers have been working a lot on finding out what causes ASD so they can develop new treatments that can help these people. Some of this research is about how the stress system works. Everyone has such a system and normally it ensures, for example, that your heart beats faster when you are afraid but also that you can calm down as soon as you are no longer afraid. In people with ASD, researchers have found that their stress system does not work quite the same as in people without ASD. However, in order to get more clarity on this, more studies would have to be executed. Therefore, we would like to ask you if you are interested in participating in this study.

You are free to choose whether you want to participate and you can always say if you no longer want to participate. If you decide to participate and your parents have also given their permission, you will be asked to carry out the following tasks:

1) Filling out questionnaires: both you and your parents will be sent some online questionnaires. You should try to complete these as completely as possible. For you, these questionnaires will take about half an hour. The questionnaires focus on the presence of symptoms from the autism spectrum, certain behavioral characteristics, various aspects related to daily functioning, physical activity and the presence of stress. If it appears that you have symptoms of the autism spectrum, you and your parents will be informed and your participation in the study will be refused. If you and your parents would like additional information, you can always contact the clinicians involved in this study (Prof. Dr. Jean Steyaert and Prof. Dr. Tine Van Damme).
2) Carrying out a stress test: this takes about an hour. The place and time of the test will be discussed in consultation with your parents. During the test, an investigator will explain everything to you gradually. This researcher will also use some sensors (small measuring instruments):
   - 3 sensors will be placed around your fingers with Velcro and/or tape
   - 1 wide elastic band will be applied around your waist
   - 3 self-adhesive sensors will be attached to your torso

   In this way, the examiner can watch your heart rate and your breathing, among other things. Finally, the examiner will also ask you three times to chew on a cotton swab so that we can collect a little saliva from you.

Benefits

If you wish to participate, you can choose from several small compensations, such as a cinema ticket or a 10-euro credit voucher (bol.com), as a thank you for your participation. If interested, you and your parents can also receive the general results of the study and your personal results after completion of the study.

If you have any questions about this study, please discuss them with your parents. If necessary, you can also send an e-mail to one of the researchers:

dra. Anoushka Thoen
anoushka.thoen@kuleuven.be

Prof. dr. Tine Van Damme
tine.vandamme@kuleuven.be

ASSENT FORM

Title of the study: Evaluating the potential of respiratory-sinus-arrhythmia biofeedback for reducing physiological stress in adolescents with autism: study protocol for a randomized controlled trial

- □ I have been allowed to choose whether or not to participate in this study.
  - □ I want to participate in this study.
  - □ I have been given the opportunity to read this letter and to ask questions when something was not clear.
  - □ It is clear to me that I may stop at any time and that I do not have to tell anyone why I no longer want to participate.
- □ I do NOT want to participate in this study.

FIRST NAME ....

SURNAME ....

DATE OF BIRTH ....

Date, name and signature:

Upon completion of this study (please indicate):

- □ I would like to receive the overall results of the study.
- □ I would like to receive the personal scores on the questionnaires.
- □ I would like to receive the personal results of the stress test.

You may send this information to the following e-mail address

....
